# The Practice of Cranial Neurosurgery and the Malpractice Liability Environment in the United States

**DOI:** 10.1371/journal.pone.0121191

**Published:** 2015-03-23

**Authors:** Kimon Bekelis, Symeon Missios, Kendrew Wong, Todd A. MacKenzie

**Affiliations:** 1 Section of Neurosurgery, Dartmouth-Hitchcock Medical Center, Lebanon, New Hampshire, United States of America; 2 Department of Neurosurgery, Cleveland Clinic Foundations, Cleveland, Ohio, United States of America; 3 The Dartmouth Institute for Health Policy and Clinical Practice, Lebanon, New Hampshire, United States of America; 4 Geisel School of Medicine at Dartmouth, Hanover, New Hampshire, United States of America; Emory University School of Medicine, UNITED STATES

## Abstract

**Object:**

The potential imbalance between malpractice liability cost and quality of care has been an issue of debate. We investigated the association of malpractice liability with unfavorable outcomes and increased hospitalization charges in cranial neurosurgery.

**Methods:**

We performed a retrospective cohort study involving patients who underwent cranial neurosurgical procedures from 2005-2010, and were registered in the National Inpatient Sample (NIS) database. We used data from the National Practitioner Data Bank (NPDB) from 2005 to 2010 to create measures of volume and size of malpractice claim payments. The association of the latter with the state-level mortality, length of stay (LOS), unfavorable discharge, and hospitalization charges for cranial neurosurgery was investigated.

**Results:**

During the study period, there were 189,103 patients (mean age 46.4 years, with 48.3% females) who underwent cranial neurosurgical procedures, and were registered in NIS. In a multivariable regression, higher number of claims per physician in a state was associated with increased ln-transformed hospitalization charges (beta 0.18; 95% CI, 0.17 to 0.19). On the contrary, there was no association with mortality (OR 1.00; 95% CI, 0.94 to 1.06). We observed a small association with unfavorable discharge (OR 1.09; 95% CI, 1.06 to 1.13), and LOS (beta 0.01; 95% CI, 0.002 to 0.03). The size of the awarded claims demonstrated similar relationships. The average claims payment size (ln-transformed) (Pearson’s rho=0.435, P=0.01) demonstrated a positive correlation with the risk-adjusted hospitalization charges but did not demonstrate a correlation with mortality, unfavorable discharge, or LOS.

**Conclusions:**

In the present national study, aggressive malpractice environment was not correlated with mortality but was associated with higher hospitalization charges after cranial neurosurgery. In view of the association of malpractice with the economics of healthcare, further research on its impact is necessary.

## Introduction

A survey by the American Medical Association (AMA) showed that 5% of physicians face a malpractice claim yearly.[1] Neurosurgery tops the malpractice list for all subspecialties, with 19.1% of neurosurgeons involved in a claim annually.[2] Recent increases in malpractice premiums and rapid growth in the number and size of awards to plaintiffs have raised widespread concerns about the medical malpractice liability system.[3, 4] Some argue that the current system plays a role in maintaining the quality of care.[4, 5] Others point out that it fails to compensate most patients who suffer avoidable injuries, and punishes many physicians for adverse events that were not caused by negligence.[4, 5] Although there is evidence for the latter in some medical specialties,[4] this paradoxical imbalance between liability environment and outcomes has not been investigated before in neurosurgery, the surgical field generating the majority of malpractice claims.[2] Concerns have also been raised[6] in regards to the effect of increasing liability on the practice of defensive medicine, resulting in increased hospitalization charges.

Several studies have attempted to characterize malpractice claims in neurosurgery.[6–12] Some of these have focused on physician surveys about defensive medicine[6, 11, 13] and others on the context and size of malpractice claims.[7, 9] Most of the literature involves retrospective analyses of single institution experiences,[7, 9] demonstrating results with limited generalization, given their inherent selection bias. The interpretation of other multi-center studies is equally limited given their focus on specific subgroup data or a specific region of the US.[10, 12] There has been no investigation of the association of the local malpractice liability environment with unfavorable outcomes and hospitalization charges.

The National Inpatient Sample (NIS) is a hospital discharge database that represents approximately 20% of all inpatient admissions to nonfederal hospitals in the United States[14]. It allows for the unrestricted study of the patient population in question. By combining data from the NIS, National Practitioner Data Bank (NPDB), and Area Resource File (ARF) we investigated the association of the volume and size of claims payments at the state-level with mortality, length of stay, unfavorable discharge, and hospitalization charges after cranial neurosurgery. The present analysis focuses only on cranial procedures, which are exclusively performed by neurosurgeons (as opposed to spinal procedures), in order to maintain a homogeneous cohort.

## Methods

### National Inpatient Sample (NIS) Database

All patients undergoing cranial neurosurgical procedures, who were registered in the National Inpatient Sample (NIS) Database[14] (Healthcare Cost and Utilization Project, Agency for Healthcare Research and Quality, Rockville, MD) between 2005 and 2010 were included in the analysis. For these years, the NIS contains discharge data regarding 100% of discharges from a stratified random sample of nonfederal hospitals in several States to approximate a representative 20% subsample of all nonfederal US hospital discharges. More information about the NIS is available at http://www.ahcpr.gov/data/hcup/nisintro.htm.

### National Practitioner Data Bank (NPDB)

We used data from the National Practitioner Data Bank (NPDB) from 2005 to 2010 to create measures of volume and magnitude of claims payments.[15] This database is maintained by the Health Resources and Services Administration, and contains approximately 200,000 medical malpractice payments made on behalf of physicians since 1990. Despite limitations (such as the “corporate shield” loophole and potential under-reporting), NPDB is the most representative national database on medical malpractice payments and the size of these potential biases is limited.[4]

### Area Resource File (ARF)

We used the Area Resource File (ARF) 2005–2010, a national county-level health information database, maintained by the United States Department of Health and Human Services, to create measures of resource availability. By combining the county data and the 2010 census data, the state density of all physicians and of neurosurgeons were calculated.

### Cohort Definition

In order to establish the cohort of patients, we used ICD-9-CM (*International Classification of Disease-9-Current Modification*) codes to identify patients in the NIS who underwent any cranial neurosurgical procedure (craniotomy for aneurysm clipping, craniotomy for tumor and AVM resection, brain biopsy, cranioplasty, shunt placement, craniotomy/burr holes for trauma, deep brain stimulation, and transphenoidal/transfrontal pituitary tumor resection) between 2005 and 2010 (S1 Table).

### Outcome Variables

The primary outcome variables were mortality, average length of stay (LOS) of hospitalization, unfavorable discharge (discharge to short- or long-term facility, other than the patient’s home), and the average hospitalization charges after cranial neurosurgical procedures. All outcome values were calculated at the state level. Although charges often involve substantial internal transfers and overhead accounting, we elected to utilize this measure instead of cost, because accurate cost values could not be reliably calculated using NIS data. Cost calculations in this database are average estimates based on cost-to-charge ratio files, which are created by averaging all-payer inpatient costs for all patients of a given hospital per year, and are not representative of the true hospitalization cost of neurosurgical patients.

### Exposure variables

The association of the outcomes with the pertinent exposure variables was examined using regression analysis. Age, modified Charlson Comorbidity Index (CCI)[16, 17] and state density of neurosurgeons (average number of neurosurgeons per 100,000 population per state) were continuous variables. Gender, race (African American, Hispanic, Asian, or other, with Caucasian being the reference value), insurance (private insurance, self pay, other insurance, Medicaid, with Medicare being the reference value), and income were categorical variables. Income was defined as the median income based on zip code, and was divided into quartiles, with the lowest quartile being the reference value.

We utilized two continuous variables reflecting state-level measures of the malpractice liability environment. The one was the mean dollar value of malpractice payments (arising from both judgments and settlements) per physician in each state. The second was the mean number of malpractice claims per 100 physicians per state. The choice of these measures was based on findings[4] that physicians respond to the number of awarded claims as well as to the average size of malpractice awards.

The hospital characteristics, used in the analysis as categorical variables, included hospital region (Northeast, Midwest, South, West with Northeast being the reference value), hospital location (rural, urban teaching, urban non-teaching, with rural being the reference value), and hospital bed size (small, medium, large, with small being the reference value). More information of the definitions of the various categories of hospital characteristics can be found at http://www.hcup-us.ahrq.gov/db/vars/nis_stratum/nisnote.jsp.

### Statistical analysis

Categorical variables were expressed in percentages. Normally distributed continuous variables were expressed using mean and standard deviation (SD), and non-normally distributed continuous variables were expressed using median and interquartile range (IQR). When comparing demographics, continuous variables were compared using Student’s t-test or Mann-Whitney test, as appropriate, and categorical variables were compared using Chi-square test. Patients with missing variables were excluded from the analysis using listwise deletion.

Examination of the distribution of values for hospitalization charges and average claims payment per physician per state revealed significant positive skewness (4.1 and 1.4 respectively). In order to normalize the distribution of these values and provide a better fit for the data, log transformation was performed using the natural logarithm (ln) of the values. After ln-transformation skewness improved to 0.3 for hospitalization charges and 0.6 for claims payments per 100 physicians per state.

The association of mortality and unfavorable discharge with the variable of interest (number of awarded claims per 100 physicians per state and ln(average claims payment per physician per state)) was examined using a multivariable logistic regression model. The association of length of stay with the exposure variables of interest was examined with a generalized linear regression model using gamma distribution, which provided better fit for the data. The association of hospitalization charges with the variables of interest was examined using a generalized linear model after ln transformation of the charges, which significantly improved fit of the data. The covariates included in all the models were age, sex, race, CCI, payment, income, state density of neurosurgeons, hospital region, hospital location, and hospital bed size. To confirm that these associations were present in individual procedures, we repeated the above regressions in the most common subgroups of procedures in our cohort (craniotomy for tumor, craniotomy for aneurysm clipping, craniotomy for trauma).

We subsequently created risk-adjusted values of the primary outcomes. Risk-adjustment was performed using a fixed effects multivariable logistic regression model for the outcome measure of mortality and unfavorable discharge and a generalized linear model using gamma distribution for the outcome of LOS and a generalized linear model for the ln-transformed hospitalization charges. The covariates included in the models were age, sex, race, CCI, payment, income, number of neurosurgeons per state, hospital region, location and bedsize. Predicted values for each outcome were obtained from the model at the individual patient level. Risk-adjusted values were then calculated by dividing the observed values over the predicted values and aggregating the individual patient data at the state level to calculate state-level predicted rates of mortality, unfavorable discharge, LOS and ln-transformed hospitalization charges. Scatter plots were created to demonstrate the correlation of the risk-adjusted values of the outcomes with the number of awarded claims and the ln-transformed claims payment size. Pearson’s rho coefficients and p-values were calculated.

Statistical analyses were performed using SPSS version 20 (IBM, Armonk, NY), XLSTAT version 2013.6.02 (Addinsoft, New York, NY). All probability values are the results of two-sided tests, and the level of significance was set at P < 0.05.

## Results

### Patient Characteristics

**Fig 1:**
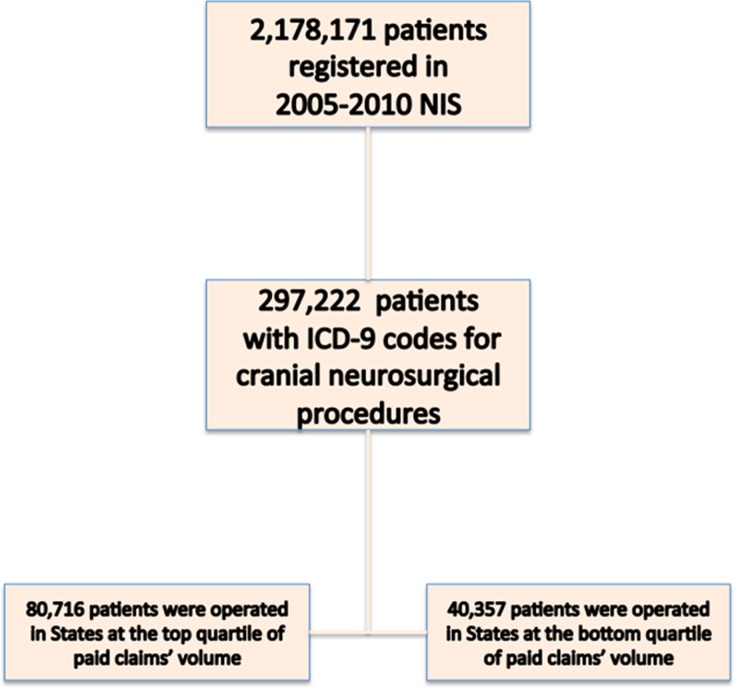
Cohort selection for the study.

In the selected study period there were 189,103 patients (Fig. 1) undergoing cranial neurosurgical procedures (mean age 46.4 years, with 48.3% females), who were registered in NIS. Of these, 48,555 were operated in states that belong to the highest quartile of claims’ volume, and 47,189 in states that belong to the lowest quartile of claims’ volume. The relative distribution of the exposure variables is demonstrated in Table 1.

**Table 1:** Patient and hospital characteristics.

		All Patients		States at the top quartile of paid claims’ volume		States at the bottom quartile of paid claims’ volume		P-Value
		**N**		**N**		**N**		
**Sample size**		189103		48555		47189		
		**Mean**	**SD**	**Mean**	**SD**	**Mean**	**SD**	
**Age**		46.4	23.7	47.9	23.7	46.2	23.4	<0.0001
		**N**	**%**	**N**	**%**	**N**	**%**	
**Sex**	F	90603	48.3	23369	48.1	23019	48.8	0.044
	M	97108	51.7	25182	51.9	24167	51.2	
	Unreported data	1392		4		3		
**Quartiles of median income based on zip code**	1^st^ Quartile	44392	24.1	12807	27.5	10330	22.3	<0.0001
	2^nd^ Quartile	45612	24.8	11423	24.5	13266	28.6	<0.0001
	3^rd^ Quartile	46899	25.5	10994	23.6	12604	27.2	<0.0001
	4^th^ Quartile	47094	25.6	11386	24.4	10106	21.8	<0.0001
	Unreported data	5106		1945		883		
**Payer**	Medicare	52297	27.7	14571	30.0	13005	27.7	<0.0001
	Medicaid	31418	16.6	8394	17.3	7098	15.1	<0.0001
	Private payer	86483	45.8	21059	43.4	22473	47.9	<0.0001
	Self-payer	9149	4.8	2102	4.3	2273	4.8	<0.0001
	Other	9387	5.0	2390	4.9	2091	4.5	<0.0001
	Unreported data	369		39		249		
**Charlson Comorbidity Index (CCI)**	Low (0–3)	158025	83.6	39996	82.4	40129	85.0	<0.0001
	Moderate/High (> = 4)	31078	16.4	8559	17.6	7060	15.0	
**Race**	Caucasian	103221	70.7	31610	70.0	19280	40.9	<0.0001
	African American	15604	10.7	5635	12.5	2712	5.7	<0.0001
	Hispanic	17047	11.7	4562	10.1	1138	2.4	<0.0001
	Asian	4178	2.9	853	1.9	607	1.3	<0.0001
	Other	6047	4.1	2506	5.5	792	1.7	<0.0001
	Unreported cases	43006		3389		22660		
**Region**	Northeast	35315	18.7	25009	51.5	**1093**	**2.3**	<0.0001
	Midwest	38080	20.1	855	1.8	16044	34.0	<0.0001
	South	68923	36.4	22691	46.7	16409	34.8	<0.0001
	West	46785	24.7	0	0	13643	28.9	<0.0001
**Location**	Rural	4318	2.3	723	1.5	1003	2.1	<0.0001
	Urban Nonteaching	34097	18.0	7617	15.7	7353	15.6	0.654
	Urban Teaching	150688	79.7	40215	82.8	38833	82.3	0.030
**Bedsize**	Small	13262	7.0	2057	4.2	4059	8.6	<0.0001
	Medium	32471	17.2	7105	14.6	7134	15.1	0.035
	Large	143370	75.8	39393	81.1	35996	76.3	<0.0001
		**Mean**	**SD**	**Mean**	**SD**	**Mean**	**SD**	
**Neurosurgeons per 100,000 population**		1.73	0.31	1.81	0.24	1.87	0.31	<0.0001

SD: Standard Deviation

### Clinical Outcomes

Nationwide mortality for cranial neurosurgical procedures in our cohort was 5.2%. The median length of stay was 5.0 days (IQR 8.0). The median hospitalization charges were $57,670 (IQR $75,212). Patients in the highest quartile of claims’ volume had a higher chance of discharge to rehabilitation, longer length of stay and produced higher hospitalization charges. (Table 2)

**Table 2:** Primary outcomes for patients undergoing cranial neurosurgical procedures in the United States.

	All Patients		Top 25% claims frequency patients		Bottom 25% claims frequency patients		P-Value
	N	%	N	%	N	%	
**Mortality**	9775	5.17	2775	5.7	2273	4.8	<0.0001
**Unfavorable discharge**	**61255**	**34.25**	**18001**	**39.4**	**14289**	**31.9**	<0.0001
	**Median**	**IQR**	**Median**	**IQR**	**Median**	**IQR**	
**Median length of stay**	5.0	8	6.0	9	5.0	8	<0.0001
**Median hospitalization charges**	57670	75212	62265	82324	48777	58060	<0.0001

### Multivariable analysis

In a multivariable regression analysis (Table 3, S2–S5 Tables), higher number of claims per 100 physicians per state was associated with increased ln-transformed hospitalization charges (beta 0.18; 95% CI, 0.17 to 0.19). On the contrary, number of claims was not associated with mortality (OR 1.00; 95% CI, 0.94 to 1.06) after cranial neurosurgical procedures. There was a very weak association with LOS (beta 0.01; 95% CI, 0.002 to 0.03), and unfavorable discharge (OR 1.09; 95% CI, 1.05 to 1.13).

**Table 3:** Regression models for primary outcomes and variables of interest.

	Number of paid claims per 100 physicians per state			Ln transformed Size of paid claims per physician per state¶		
	OR	95% CI	P value	OR	95% CI	P value
**Mortality** ^**1**^	1.00	0.94–1.06	0.974	1.05	0.97–1.13	0.219
**Unfavorable discharge** ^**1**^	1.09	1.05–1.13	<0.0001	1.09	1.05–1.13	<0.0001
**Length of stay** ^**2**^ ^**§**^	0.01	0.002–0.03	0.024	0.04	0.03–0.06	<0.0001
**Ln transformed Hospitalization charges** ^**3**^ ^**§**^	0.18	0.17–0.19	<0.0001	0.12	0.11–0.14	<0.0001

OR: Odds Ratio; 95% CI: 95% Confidence Interval

¶The payment amount was ln transformed to provide a better fit for the data

^1^Based on a logistic regression model including age, sex, income, payer, CCI, race, hospital region, hospital location, hospital bedsize, density of neurosurgeons as covariates

^2^Based on a generalized linear model including age, sex, income, payer, CCI, race, hospital region, hospital location, hospital bedsize, density of neurosurgeons as covariates

^3^Based on a generalized linear model including age, sex, income, payer, CCI, race, hospital region, hospital location, hospital bedsize, density of neurosurgeons as covariates. Hospitalization charges underwent an ln transformation because this provided the best fit for our data

§The point estimates from these linear regressions represent beta coefficients and not odds ratios

Similarly (Table 3, S6–S9 Table) increased size of claims awarded per physician per state (ln transformed for better fit of data) was associated with increased ln-transformed hospitalization charges (beta 0.12; 95% CI, 0.11 to 0.14), but demonstrated no association with mortality (OR 1.05; 95% CI, 0.97 to 1.13), after cranial neurosurgical procedures. There was a very weak association with LOS (beta 0.04; 95% CI, 0.03 to 0.06), and unfavorable discharge (OR 1.09; 95% CI, 1.05 to 1.13).

When examining the subgroups of the most frequently performed procedures in our cohort, we identified similar associations (Table 4).

**Table 4:** Regression models for primary outcomes and variables of interest in the 3 most common procedure subgroups.

Craniotomy for tumor						
	Number of paid claims per 100 physicians per state			Ln transformed Size of paid claims per physician per state¶		
	OR	95% CI	P value	OR	95% CI	P value
**Mortality** ^**1**^	1.11	0.98–1.26	0.101	1.05	0.90–1.24	0.513
**Unfavorable discharge** ^**1**^	1.13	1.08–1.18	<0.0001	1.08	1.01–1.14	0.023
**Length of stay** ^**2**^ ^**§**^	0.01	0.001–0.03	0.046	0.02	-0.002–0.04	0.076
**Ln transformed Hospitalization charges** ^**3**^ ^**§**^	0.16	0.15–0.18	<0.0001	0.08	0.06–0.10	<0.0001
**Craniotomy for aneurysm clipping**						
	**Number of paid claims per 100 physicians per state**			**Ln transformed Size of paid claims per physician per state¶**		
	**OR**	**95% CI**	**P value**	**OR**	**95% CI**	**P value**
**Mortality** ^**1**^	1.02	0.79–1.32	0.864	1.03	0.74–1.43	0.870
**Unfavorable discharge** ^**1**^	1.30	1.14–1.48	<0.0001	1.32	1.12–1.57	0.001
**Length of stay** ^**2**^ ^**§**^	-0.01	-0.06–0.04	0.646	0.04	-0.03–0.10	0.237
**Ln transformed Hospitalization charges** ^**3**^ ^**§**^	0.22	0.18–0.27	<0.0001	0.21	0.15–0.26	<0.0001
**Craniotomy for trauma**						
	**Number of paid claims per 100 physicians per state**			**Ln transformed Size of paid claims per physician per state¶**		
	**OR**	**95% CI**	**P value**	**OR**	**95% CI**	**P value**
**Mortality** ^**1**^	1.08	0.93–1.26	0.302	1.27	0.97–1.55	0.240
**Unfavorable discharge** ^**1**^	1.04	0.95–1.15	0.419	1.02	0.90–1.16	0.712
**Length of stay** ^**2**^ ^**§**^	0.04	0.001–0.08	0.042	0.09	0.04–0.13	0.001
**Ln transformed Hospitalization charges** ^**3**^ ^**§**^	0.20	0.16–0.24	<0.0001	0.13	0.08–0.17	<0.0001

OR: Odds Ratio; 95% CI: 95% Confidence Interval

¶The payment amount was ln transformed to provide a better fit for the data

^1^Based on a logistic regression model including age, sex, income, payer, CCI, race, hospital region, hospital location, hospital bedsize, density of neurosurgeons as covariates

^2^Based on a generalized linear model including age, sex, income, payer, CCI, race, hospital region, hospital location, hospital bedsize, density of neurosurgeons as covariates

^3^Based on a generalized linear model including age, sex, income, payer, CCI, race, hospital region, hospital location, hospital bedsize, density of neurosurgeons as covariates. Hospitalization charges underwent an ln transformation because this provided the best fit for our data

§The point estimates from these linear regressions represent beta coefficients and not odds ratios

### Correlations for risk-adjusted outcomes

**Fig 2:**
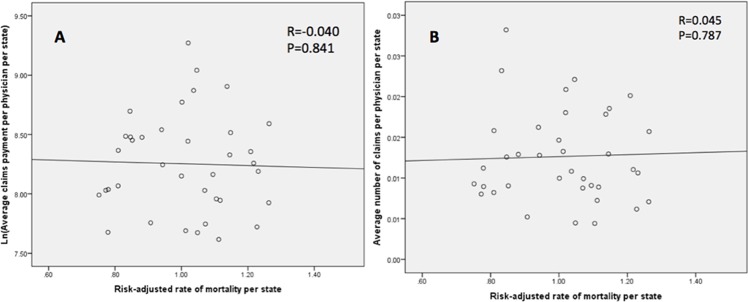
Scatter plot demonstrating the correlation of average state-level mortality for cranial neurosurgical procedures and average size (ln-transformed) (A) and number (B) of paid claims per physician per state.

**Fig 3:**
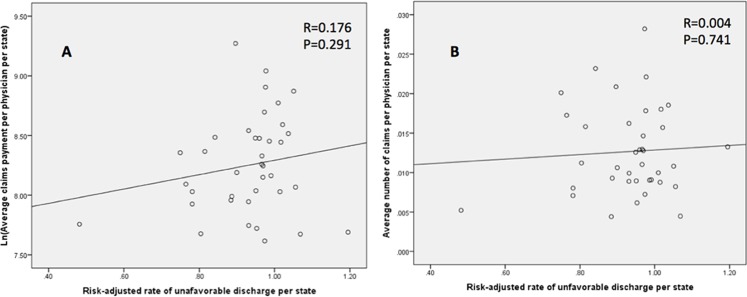
Scatter plot demonstrating the correlation of average state-level length of stay for cranial neurosurgical procedures and average size (ln-transformed) (A) and number (B) of paid claims per physician per state.

**Fig 4:**
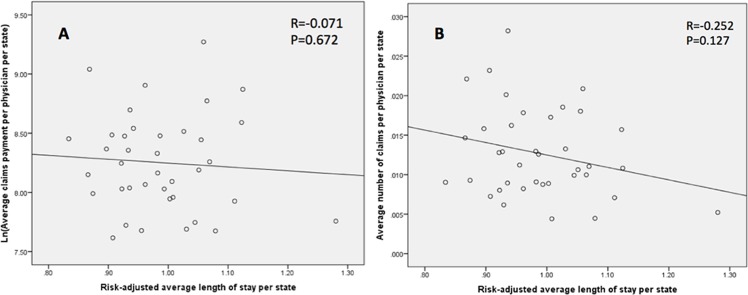
Scatter plot demonstrating the correlation of the rate of unfavorable discharge after cranial neurosurgical procedures and average size (ln-transformed) (A) and number (B) of paid claims per physician per state.

**Fig 5:**
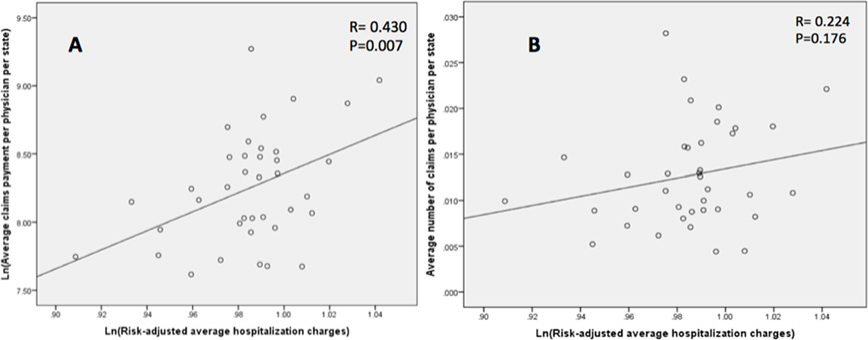
Scatter plot demonstrating the correlation of the ln-transformed average state-level hospitalization charges for cranial neurosurgical procedures and average size (ln-transformed) (A) and number (B) of paid claims per physician per state.

Our primary outcomes were risk-adjusted and their correlation with the size and volume of malpractice claims was examined at the state level. Higher number of claims per 100 physicians per state did not demonstrate a significant correlation with mortality (Pearson’s rho = 0.045, P = 0.787) (Fig. 2B), unfavorable discharge (Pearson’s rho = 0.004, P = 0.741) (Fig. 3B), or LOS (Pearson’s rho = -0.252, P = 0.127) (Fig. 4B). Likewise, increased size of claims awarded per physician per state (ln transformed) was not correlated with mortality (Pearson’s rho = 0.040, P = 0.841) (Fig. 2A), unfavorable discharge (Pearson’s rho = 0.176, P = 0.291) (Fig. 3B), or LOS (Pearson’s rho = -0.071, P = 0.672) (Fig. 4A). On the other hand, the size of paid malpractice claims (ln-transformed) demonstrated (Fig. 5A) a positive correlation with ln-transformed hospitalization charges (Pearson’s rho = 0.430, P = 0.007) after cranial neurosurgery. The volume of malpractice claims (Fig. 5B) also demonstrated a positive correlation, although not statistically significant. (Pearson’s rho = 0.224, P = 0.176)

## Discussion

An environment of increased malpractice liability was not associated with higher mortality but was related to rising hospitalization charges among patients undergoing cranial neurosurgical procedures, in this large population level study. We utilized the number and size of paid claims as surrogates of the state-level malpractice environment. Prior research[4] has identified these factors as some of the most important drivers of the perceived threat of malpractice among physicians. Although physicians can be protected against indemnity payments through malpractice insurance, they cannot insure against the indirect cost of litigation, such as time, stress, added work, and reputational damage.[4] This is particularly important for neurosurgeons, who face the most hostile liability environment among the surgical specialties.[2] In this setting, the study of the association of litigation with negative outcomes and its impact on the practice of neurosurgery is of major significance.

Mortality after cranial neurosurgical procedures was not associated with an aggressive malpractice environment. The paradoxical imbalance between death and litigation is present in all procedure subgroups, and has been detected before in other medical specialties.[4] In the same direction, several other studies have demonstrated a disconnect between negligence and litigation.[5, 18, 19] Studdert et al[20] have shown that nearly 40% of claims were not associated with medical errors. In addition, the Harvard Malpractice Study[5] and the California Medical Association’s Medical Insurance feasibility study[21] found very low negligence rates for adverse events, ranging from 0.8–1%. In the present study, there was only a mild, but inconsistent across subgroups, association of LOS and unfavorable discharge with malpractice claims. Although it is possible that complications resulting in these negative outcomes could drive litigation, these associations did not persist when performing state-level risk adjusted correlations. It appears that local factors were the most significant drivers of the malpractice environment.

Despite the lack of association of litigation with mortality in cranial neurosurgery, the former was associated with increased hospitalization charges. Several studies have identified the practice of defensive medicine, resulting in more procedures and tests ordered during the postoperative hospitalization period, as the major contributor to this phenomenon.[6, 11, 13] It is not clear from the present study whether the observed associations are the result of defensive medicine. Although the etiology of this phenomenon cannot be identified through observational data, the association of neurosurgery hospitalization charges with aggressive malpractice environment warrants further investigation. Especially in the context of the national efforts for cost containment, malpractice claims, although currently not receiving much attention, can become a target for cost optimization, especially considering the staggering amount of money paid in malpractice claims, rising to $1.5 billion in 2009.[22]

In neurosurgery, in which the average time to resolution of a claim is 22.3 months and the mean annual probability of a malpractice claim is 18.9%, it has been estimated that nearly 11 years (27.2%) of a surgeon’s career is spent with a malpractice claim outstanding.[23] In addition to this cost for providers at a personal level, the economic burden of the current malpractice system to physicians and society is extremely heavy, especially considering the liability-outcomes dissociation. Our study provides limited guidance on the potential impact of malpractice reform on the practice of neurosurgery. From a clinical perspective, it is important to recognize that this analysis does not address the question of how the amount of care for an individual patient in a specific case would affect the patient’s clinical outcome and the possibility of litigation. From a policy perspective, our study does not indicate whether it is possible to reduce overutilization and spending without affecting patient outcomes. However, if the United States as a whole could safely achieve a litigation environment similar to the lowest-utilizing areas, significant savings could be achieved. Further research in that direction is needed.

The present study has several limitations common to administrative databases. Indication bias and residual confounding could account for some of the observed associations. In addition, some coding inaccuracies will undoubtedly occur and can affect our estimates. This is no different than other studies involving the NIS. The NIS during the years studied did not include hospitals from all states. However, the creation of the 20% random sample to be included in NIS is done in such a way by the HCUP that the hospitals included are still representative of the nation. Although NIS provides accurate data on hospitalization charges, reliable calculation of cost was not possible using this database. In addition, some data categories were not available for all patients. To avoid the introduction of further bias we excluded those patients from any analysis. Additionally, we were lacking post-hospitalization, and long-term data on these patients, as well as disease severity. However, since two thirds of paid claims involve death,[15] we believe that the use of mortality as a metric is representative. Lastly, we are analyzing the observed associations but causality is very hard to establish based on ecologic data.

An important limitation of the NPDB is the inability to analyze claims that do not result in payments. However, the literature supports that paid claims are the most important determinants of perceived aggressiveness of the litigation system.[15] In addition, the NPDB underestimates the number of malpractice payments because settlements paid on behalf of corporate entities instead of physicians are exempt from reporting.[24] However, this phenomenon is not expected to be unevenly distributed throughout the US.[22]

## Conclusions

Neurosurgeons are faced with a large number of malpractice claims during their careers. The potential imbalance between malpractice liability cost and quality of care has been an issue of debate in several areas of medicine. In the present national study, merging 3 large databases (NIS, NPDB, ARF), aggressive malpractice environment was not correlated with mortality but was associated with higher hospitalization charges after cranial neurosurgery. In view of the association of malpractice with the economics of healthcare, further research on its impact is necessary.

## Supporting Information

S1 Table Coding definitions. (DOC)

S2 Table Regression models demonstrating the association of exposure variables (variable of interest: number of claims per 100 physicians per state) with length of hospitalization of patients undergoing cranial neurosurgical procedures. (DOC)

S3 Table Regression model demonstrating the association of exposure variables (variable of interest: number of claims per 100 physicians per state) with ln transformed hospitalization charges of patients undergoing cranial neurosurgical procedures. (DOC)

S4 Table Regression model demonstrating the association of exposure variables (variable of interest: number of claims per 100 physicians per state) with in-hospital mortality of patients undergoing cranial neurosurgical procedures. (DOC)

S5 Table Regression model demonstrating the association of exposure variables (variable of interest: number of claims per 100 physicians per state) with unfavorable discharge of patients undergoing cranial neurosurgical procedures. (DOC)

S6 Table Regression model demonstrating the association of exposure variables (variable of interest: ln transformed average claims payments per physician per state) with length of hospitalization of patients undergoing cranial neurosurgical procedures. (DOC)

S7 Table Regression model demonstrating the association of exposure variables (variable of interest: ln transformed average claims payments per physician per state) with ln transformed hospitalization charges of patients undergoing cranial neurosurgical procedures. (DOC)

S8 Table Regression model demonstrating the association of exposure variables (variable of interest: ln transformed average claims payments per physician per state) with in-hospital mortality of patients undergoing cranial neurosurgical procedures. (DOC)

S9 Table Regression model demonstrating the association of exposure variables (variable of interest: ln transformed average claims payments per physician per state) with unfavorable discharge of patients undergoing cranial neurosurgical procedures. (DOC)
