# Supplementary material for: The Practice of Cranial Neurosurgery and the Malpractice Liability Environment in the United States
Source: PLoS One. 2015 Mar 23;10(3):e0121191. doi: 10.1371/journal.pone.0121191 (PMC4370383; doi:10.1371/journal.pone.0121191)
Supplement: S1 Table — (DOC) [file pone.0121191.s001.doc]

| **S1 Table. Coding definitions** | | |
| --- | --- | --- |
| **GROUP** | **CATEGORY** | **ICD-9 PROCEDURE CODES** |
| **Procedures** | **Craniotomy for aneurysm clipping** | **39.51** |
|  | **Craniotomy for tumor, epilepsy and AVM resection** | **01.53, 01.59, 01.51** |
|  | **Brain biopsy** | **01.11, 01.12, 01.13, 01.14** |
|  | **Craniotomy/burr hole for trauma** | **01.2, 01.21, 01.22, 01.23, 01.24, 01.25, 01.26, 01.27,**  **01.28, 02.02** |
|  | **Shunt Placement** | **02.2, 02.32, 02.33, 02.34, 02.35, 02.39, 03.7, 03.71, 03.72, 03.79, 02.4, 02.41, 02.42, 02.43** |
|  | **Deep brain stimulation** | **02.93, 01.22** |
|  | **Cranioplasty** | **02.0, 02.05, 02.07** |
|  | **Hypophysectomy** | **07.61, 07.62, 07.64, 07.65** |
