# Supplementary material for: The Practice of Cranial Neurosurgery and the Malpractice Liability Environment in the United States
Source: PLoS One. 2015 Mar 23;10(3):e0121191. doi: 10.1371/journal.pone.0121191 (PMC4370383; doi:10.1371/journal.pone.0121191)
Supplement: S4 Table — (DOC) [file pone.0121191.s004.doc]

**S4 Table. Regression model* demonstrating the association of exposure variables (variable of interest after rescaling: number of claims per 100 physicians per state) with in-hospital mortality of patients undergoing cranial neurosurgical procedures**

| Variable |  | OR | 95% Confidence Interval | | p value |
| --- | --- | --- | --- | --- | --- |
|  |  |  | Lower | Upper |  |
| Number of claims per 100 physicians per state |  | 1.00 | 0.95 | 1.06 | 0.974 |
| Age |  | 1.02 | 1.02 | 1.02 | <0.0001 |
| CCI |  | 1.02 | 1.01 | 1.03 | <0.0001 |
| Neurosurgeons per 100,000 population per state |  | 0.92 | 0.84 | 1.00 | 0.062 |
| Gender | F | 0.75 | 0.72 | 0.79 | <0.0001 |
|  | M | Ref |  |  |  |
| Region | West | 0.95 | 0.87 | 1.04 | 0.302 |
|  | South | 1.00 | 0.93 | 1.08 | 0.965 |
|  | Midwest | 0.93 | 0.84 | 1.02 | 0.136 |
|  | Northeast | Ref |  |  |  |
| Location | Urban teaching | 0.99 | 0.85 | 1.15 | 0.872 |
|  | Urban non-teaching | 1.11 | 0.95 | 1.31 | 0.193 |
|  | Rural | Ref |  |  |  |
| Bedsize | Large | 1.83 | 1.61 | 2.08 | <0.0001 |
|  | Medium | 1.82 | 1.58 | 2.08 | <0.0001 |
|  | Small | Ref |  |  |  |
| Payer | Other | 1.63 | 1.47 | 1.82 | <0.0001 |
|  | Self-payer | 2.51 | 2.28 | 2.76 | <0.0001 |
|  | Private payer | 0.93 | 0.87 | 1.00 | 0.035 |
|  | Medicaid | 1.34 | 1.22 | 1.46 | <0.0001 |
|  | Medicare | Ref |  |  |  |
| Race | Other | 1.43 | 1.28 | 1.61 | <0.0001 |
|  | Asian | 1.77 | 1.56 | 2.00 | <0.0001 |
|  | Hispanic | 1.12 | 1.03 | 1.21 | 0.005 |
|  | African American | 1.64 | 1.53 | 1.76 | <0.0001 |
|  | Caucasian | Ref |  |  |  |
| Income | 4^th^ quartile | 0.77 | 0.72 | 0.83 | <0.0001 |
|  | 3^rd^ quartile | 0.85 | 0.79 | 0.90 | <0.0001 |
|  | 2^nd^ quartile | 0.84 | 0.79 | 0.90 | <0.0001 |
|  | 1^st^ quartile | Ref |  |  |  |
|  |  |  |  |  |  |

*Logistic regression model
