# Supplementary material for: The Practice of Cranial Neurosurgery and the Malpractice Liability Environment in the United States
Source: PLoS One. 2015 Mar 23;10(3):e0121191. doi: 10.1371/journal.pone.0121191 (PMC4370383; doi:10.1371/journal.pone.0121191)
Supplement: S5 Table — (DOC) [file pone.0121191.s005.doc]

**S5 Table. Regression model* demonstrating the association of exposure variables (variable of interest after rescaling: number of claims per 100 physicians per state) with unfavorable discharge of patients undergoing cranial neurosurgical procedures**

| Variable |  | OR | 95% Confidence Interval | | p value |
| --- | --- | --- | --- | --- | --- |
|  |  |  | Lower | Upper |  |
| Number of claims per 100 physicians per state |  | 1.09 | 1.06 | 1.13 | <0.0001 |
| Age |  | 1.02 | 1.02 | 1.03 | <0.0001 |
| CCI |  | 1.09 | 1.09 | 1.10 | <0.0001 |
| Neurosurgeons per 100,000 population per state |  | 1.04 | 0.99 | 1.09 | 0.102 |
| Gender | F | 0.93 | 0.91 | 0.95 | <0.0001 |
|  | M | Ref |  |  |  |
| Region | West | 0.64 | 0.61 | 0.67 | <0.0001 |
|  | South | 0.60 | 0.58 | 0.63 | <0.0001 |
|  | Midwest | 0.75 | 0.71 | 0.78 | <0.0001 |
|  | Northeast | Ref |  |  |  |
| Location | Urban teaching | 1.14 | 1.05 | 1.23 | 0.001 |
|  | Urban non-teaching | 1.48 | 1.37 | 1.61 | <0.0001 |
|  | Rural | Ref |  |  |  |
| Bedsize | Large | 1.22 | 1.16 | 1.28 | <0.0001 |
|  | Medium | 1.32 | 1.25 | 1.40 | <0.0001 |
|  | Small | Ref |  |  |  |
| Payer | Other | 0.67 | 0.63 | 0.72 | <0.0001 |
|  | Self-payer | 0.51 | 0.47 | 0.54 | <0.0001 |
|  | Private payer | 0.59 | 0.57 | 0.61 | <0.0001 |
|  | Medicaid | 0.91 | 0.86 | 0.95 | <0.0001 |
|  | Medicare | Ref |  |  |  |
| Race | Other | 1.20 | 1.13 | 1.28 | <0.0001 |
|  | Asian | 1.37 | 1.27 | 1.47 | <0.0001 |
|  | Hispanic | 1.05 | 1.01 | 1.10 | 0.014 |
|  | African American | 1.56 | 1.50 | 1.62 | <0.0001 |
|  | Caucasian | Ref |  |  |  |
| Income | 4^th^ quartile | 0.91 | 0.88 | 0.94 | <0.0001 |
|  | 3^rd^ quartile | 0.94 | 0.91 | 0.98 | 0.002 |
|  | 2^nd^ quartile | 0.95 | 0.92 | 0.99 | 0.009 |
|  | 1^st^ quartile | Ref |  |  |  |
|  |  |  |  |  |  |

*Logistic regression model
