# Supplementary material for: The Practice of Cranial Neurosurgery and the Malpractice Liability Environment in the United States
Source: PLoS One. 2015 Mar 23;10(3):e0121191. doi: 10.1371/journal.pone.0121191 (PMC4370383; doi:10.1371/journal.pone.0121191)
Supplement: S7 Table — (DOC) [file pone.0121191.s007.doc]

**S7 Table. Regression model* demonstrating the association of exposure variables (variable of interest: ln transformed average claims payments per physician per state) with ln transformed hospitalization charges of patients undergoing cranial neurosurgical procedures**

| Variable |  | OR | 95% Confidence Interval | | p value |
| --- | --- | --- | --- | --- | --- |
|  |  |  | Lower | Upper |  |
| Ln (Average claims payments per physician per state) |  | 0.12 | 0.11 | 0.14 | <0.0001 |
| Age |  | 2.08E-03 | 1.82E-03 | 2.34E-03 | <0.0001 |
| CCI |  | 0.05 | 0.04 | 0.05 | <0.0001 |
| Neurosurgeons per 100,000 population per state |  | -0.44 | -0.46 | -0.42 | <0.0001 |
| Gender | F | -0.06 | -0.07 | -0.05 | <0.0001 |
|  | M | Ref |  |  |  |
| Region | West | 0.33 | 0.31 | 0.35 | <0.0001 |
|  | South | -0.07 | -0.09 | -0.06 | <0.0001 |
|  | Midwest | -0.02 | -0.04 | 0.00 | 0.078 |
|  | Northeast | Ref |  |  |  |
| Location | Urban teaching | 0.22 | 0.19 | 0.25 | <0.0001 |
|  | Urban non-teaching | 0.16 | 0.12 | 0.19 | <0.0001 |
|  | Rural | Ref |  |  |  |
| Bedsize | Large | 0.24 | 0.22 | 0.26 | <0.0001 |
|  | Medium | 0.16 | 0.14 | 0.18 | <0.0001 |
|  | Small | Ref |  |  |  |
| Payer | Other | 0.23 | 0.21 | 0.25 | <0.0001 |
|  | Self-payer | 0.17 | 0.15 | 0.20 | <0.0001 |
|  | Private payer | 0.10 | 0.08 | 0.11 | <0.0001 |
|  | Medicaid | 0.22 | 0.20 | 0.24 | <0.0001 |
|  | Medicare | Ref |  |  |  |
| Race | Other | 0.18 | 0.15 | 0.20 | <0.0001 |
|  | Asian | 0.23 | 0.20 | 0.26 | <0.0001 |
|  | Hispanic | 0.22 | 0.20 | 0.23 | <0.0001 |
|  | African American | 0.24 | 0.22 | 0.26 | <0.0001 |
|  | Caucasian | Ref |  |  |  |
| Income | 4^th^ quartile | -0.01 | -0.02 | 0.01 | 0.442 |
|  | 3^rd^ quartile | -0.03 | -0.05 | -0.02 | <0.0001 |
|  | 2^nd^ quartile | -0.04 | -0.05 | -0.02 | <0.0001 |
|  | 1^st^ quartile | Ref |  |  |  |
|  |  |  |  |  |  |

*Generalized linear regression model
