# Supplementary material for: The Practice of Cranial Neurosurgery and the Malpractice Liability Environment in the United States
Source: PLoS One. 2015 Mar 23;10(3):e0121191. doi: 10.1371/journal.pone.0121191 (PMC4370383; doi:10.1371/journal.pone.0121191)
Supplement: S8 Table — (DOC) [file pone.0121191.s008.doc]

**S8 Table. Regression model* demonstrating the association of exposure variables (variable of interest: ln transformed average claims payments per physician per state) with in-hospital mortality of patients undergoing cranial neurosurgical procedures**

| Variable |  | OR | 95% Confidence Interval | | p value |
| --- | --- | --- | --- | --- | --- |
|  |  |  | Lower | Upper |  |
| Ln (Average claims payments per physician per state) |  | 1.05 | 0.97 | 1.13 | 0.219 |
| Age |  | 1.02 | 1.01 | 1.02 | <0.0001 |
| CCI |  | 1.02 | 1.01 | 1.03 | <0.0001 |
| Neurosurgeons per 100,000 population per state |  | 0.92 | 0.84 | 1.00 | 0.052 |
| Gender | F | 0.76 | 0.72 | 0.79 | <0.0001 |
|  | M | Ref |  |  |  |
| Region | West | 1.00 | 0.89 | 1.11 | 0.962 |
|  | South | 1.04 | 0.95 | 1.14 | 0.431 |
|  | Midwest | 0.96 | 0.86 | 1.06 | 0.434 |
|  | Northeast | Ref |  |  |  |
| Location | Urban teaching | 0.99 | 0.85 | 1.16 | 0.925 |
|  | Urban non-teaching | 1.12 | 0.95 | 1.32 | 0.173 |
|  | Rural | Ref |  |  |  |
| Bedsize | Large | 1.82 | 1.60 | 2.07 | <0.0001 |
|  | Medium | 1.80 | 1.57 | 2.07 | <0.0001 |
|  | Small | Ref |  |  |  |
| Payer | Other | 1.63 | 1.47 | 1.82 | <0.0001 |
|  | Self-payer | 2.51 | 2.28 | 2.77 | <0.0001 |
|  | Private payer | 0.93 | 0.87 | 0.99 | 0.030 |
|  | Medicaid | 1.34 | 1.22 | 1.46 | <0.0001 |
|  | Medicare | Ref |  |  |  |
| Race | Other | 1.43 | 1.27 | 1.60 | <0.0001 |
|  | Asian | 1.78 | 1.57 | 2.01 | <0.0001 |
|  | Hispanic | 1.12 | 1.04 | 1.22 | 0.004 |
|  | African American | 1.64 | 1.53 | 1.77 | <0.0001 |
|  | Caucasian | Ref |  |  |  |
| Income | 4^th^ quartile | 0.77 | 0.72 | 0.83 | <0.0001 |
|  | 3^rd^ quartile | 0.85 | 0.79 | 0.91 | <0.0001 |
|  | 2^nd^ quartile | 0.84 | 0.79 | 0.90 | <0.0001 |
|  | 1^st^ quartile | Ref |  |  |  |
|  |  |  |  |  |  |

*Logistic regression model
